# GraphClust2: Annotation and discovery of structured RNAs with scalable and accessible integrative clustering

**DOI:** 10.1093/gigascience/giz150

**Published:** 2019-12-06

**Authors:** Milad Miladi, Eteri Sokhoyan, Torsten Houwaart, Steffen Heyne, Fabrizio Costa, Björn Grüning, Rolf Backofen

**Affiliations:** 1 Bioinformatics Group, Department of Computer Science, University of Freiburg, Georges-Koehler-Allee 106, 79110 Freiburg, Germany; 2 Institute of Medical Microbiology and Hospital Hygiene, University of Dusseldorf, Universitaetsstr. 1, 40225 Dusseldorf, Germany; 3 Max Planck Institute of Immunobiology and Epigenetics, Freiburg, Stuebeweg 51, 79108 Freiburg, Germany; 4 Department of Computer Science, University of Exeter, North Park Road, EX4 4QF Exeter, UK; 5 ZBSA Centre for Biological Systems Analysis, University of Freiburg, Hauptstr. 1, 79104 Freiburg, Germany; 6 Signalling Research Centres BIOSS and CIBSS, University of Freiburg, Schaenzlestr. 18, 79104 Freiburg, Germany

**Keywords:** RNA secondary structure, structure-based clustering of RNAs, non-coding RNA annotation and discovery, comparative RNA analysis, structure conservation analysis of long RNAs, RNA structure prediction for CLIP data

## Abstract

**Background:**

RNA plays essential roles in all known forms of life. Clustering RNA sequences with common sequence and structure is an essential step towards studying RNA function. With the advent of high-throughput sequencing techniques, experimental and genomic data are expanding to complement the predictive methods. However, the existing methods do not effectively utilize and cope with the immense amount of data becoming available.

**Results:**

Hundreds of thousands of non-coding RNAs have been detected; however, their annotation is lagging behind. Here we present GraphClust2, a comprehensive approach for scalable clustering of RNAs based on sequence and structural similarities. GraphClust2 bridges the gap between high-throughput sequencing and structural RNA analysis and provides an integrative solution by incorporating diverse experimental and genomic data in an accessible manner via the Galaxy framework. GraphClust2 can efficiently cluster and annotate large datasets of RNAs and supports structure-probing data. We demonstrate that the annotation performance of clustering functional RNAs can be considerably improved. Furthermore, an off-the-shelf procedure is introduced for identifying locally conserved structure candidates in long RNAs. We suggest the presence and the sparseness of phylogenetically conserved local structures for a collection of long non-coding RNAs.

**Conclusions:**

By clustering data from 2 cross-linking immunoprecipitation experiments, we demonstrate the benefits of GraphClust2 for motif discovery under the presence of biological and methodological biases. Finally, we uncover prominent targets of double-stranded RNA binding protein Roquin-1, such as BCOR’s 3′ untranslated region that contains multiple binding stem-loops that are evolutionary conserved.

## Background

High-throughput RNA sequencing and computational screens have discovered hundreds of thousands of non-coding RNAs (ncRNAs) with putative cellular functionality [1–7]. Functional analysis and validation of this vast amount of data demand a reliable and scalable annotation system for the ncRNAs, which is currently still lacking for several reasons. First, it is often challenging to find homologs even for many validated functional ncRNAs because sequence similarities can be very low. Second, the concept of conserved domains, which is quite successfully applied for annotating proteins, is not well established for RNAs.

For many ncRNAs and regulatory elements in messenger RNAs (mRNAs), however, it is well known that the secondary structure is better conserved than the sequence, indicating the paramount importance of structure for the functionality. This fact has promoted annotation approaches that try to detect structural homologs in the forms of RNA “families” and “classes” [8]. Members of an RNA family are similar and typically stem from a common ancestor, while RNA classes combine ncRNAs that overlap in function and structure. A prominent example of an RNA class whose members share a common function without a common origin is microRNA. One common approach to detect ncRNAs of the same class is to align them first by sequence, then predict and detect functionally conserved structures by applying approaches such as RNAalifold [9], RNAz [10], or Evofold [11]. A large portion of ncRNAs from the same RNA class, however, have a sequence identity of <70%. In this sequence identity range, sequence-based alignments are not sufficiently accurate [12, 13]. Alternatively, approaches for simultaneous alignment and folding of RNAs such as Foldalign, Dynalign, and LocARNA [14–16] yield better accuracy.

Clusters of ncRNAs with a conserved secondary structure are promising candidates for defining RNA families or classes. To detect RNA families and classes, Will et al. [17] and Havgaard et al. [14] independently proposed to use the sequence-structure alignment scores between all input sequence pairs to perform hierarchical clustering of putatively functional RNAs. However, their applicability is restricted by the input size, owing to the high quartic computational complexity of the alignment calculations over a quadratic number of pairs. Albeit the complexity of similarity computation by pairwise sequence-structure alignment can be reduced to quadratic *O*(*n*^2^) of the sequence length [18], it is still infeasible for most practical purposes with several thousand sequence pairs. For scenarios of this scale, alignment-free approaches such as GraphClust [19] and Nofold [20] propose solutions.

A stochastic context-free grammar (SCFG), also known as covariance model (CM), encodes the sequence and structure features of a family in a probabilistic profile. CM-based approaches have been extensively used, e.g., for discovering homologs of known families [21] or comparing 2 families [22]. Profile-based methods [20, 23] such as Nofold generally rely on a CM database of known families to annotate and cluster sequences by comparing against the profiles; therefore, their applicability for *de novo* family or motif discovery is affected by the characteristics of the already known families and the provided models.

The GraphClust methodology uses a graph kernel approach to integrate both sequence and structure information into high-dimensional sparse feature vectors. These vectors are then rapidly clustered, with a linear-time complexity over the number of sequences, using a locality-sensitive hashing technique. While this solved the theoretical problem, the use case guiding the development of the original GraphClust work, here as GraphClust1, was tailored for a user with in-depth experience in RNA bioinformatics who already has the set of processed sequences at hand and now wants to detect RNA family and classes in this set. However, with the increasing amount of sequencing and genomic data, the tasks of detecting RNA family or classes and motif discovery have been broadened and are becoming a standard as well as appealing tasks for the analysis of high-throughput sequencing data.

To answer these demands, here we propose GraphClust2 as a full-fledged solution within the Galaxy framework [24]. With the development of GraphClust2, we have materialized the following goals; GraphClust2 is (i) allowing a smooth and seamless integration of high-throughput experimental data and genomic information; (ii) deployable by end users who are less experienced with the field of RNA bioinformatics; (iii) easily expandable for up- and downstream analysis, and allowing for enhanced interoperability; (iv) allowing for accessible, reproducible, and scalable analysis; and (v) allowing for efficient parallelizations over different platforms. To assist the end users, we have developed auxiliary data-processing workflows and integrated alternative prediction tools. The results are presented with intuitive visualizations and information about the clustering.

We show that the proposed solution has an improved clustering quality in the benchmarks. We show the applicability of GraphClust2 in some sought-after and prevailing domain scenarios. GraphClust2 supports structure-probing (SP) data such as from SHAPE (selective 2′-hydroxyl acylation analyzed by primer extension) and dimethyl sulfate (DMS) experiments. We demonstrate that the SP information assists in the clustering procedure and enhances the quality. By clustering ncRNAs from *Arabidopsis thaliana* with genome-wide *in vivo* DMS-seq data, we demonstrate that the genome-wide probing data can in practice be used for homologous discovery, beyond singleton structure predictions. Furthermore, an off-the-shelf procedure is introduced to identify locally conserved structure candidates from deep genomic alignments, by starting from a custom genomic locus. By applying this methodology to a couple of well-studied long non-coding RNAs (lncRNAs), we suggest the presence and the sparseness of local structures with highly reliable structural alignments. GraphClust2 can be used as a structure motif finder to identify the precise structural preferences of RNA binding proteins (RBPs) in cross-linking immunoprecipitation (CLIP) data. By comparing public CLIP data from 2 double-stranded RBPs, SLBP and Roquin-1, we demonstrate the advantage of a scalable approach for discovering structured elements. Under subjective binding preferences of Roquin-1 and the protocol biases, a scaled clustering uncovers structured targets of Roquin-1 that are evolutionary conserved. Finally, we propose BCOR’s mRNA as a prominent binding target of Roquin-1 that contains multiple stem-loop binding elements.

## Materials and Methods

### Methods overview

#### The clustering workflow

**Figure 1:**
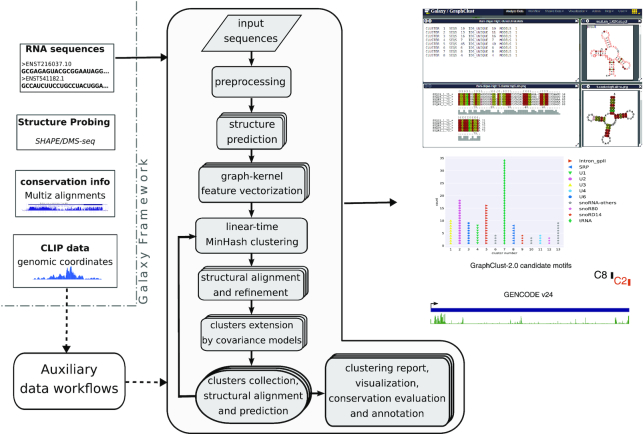
Overview of the GraphClust2 methodology. The flow chart represents the major clustering steps and is supplemented by graphical representations of the associated output data entries. The dashed arrows indicate optional data paths. Auxiliary workflows facilitate integrative clustering of experimental and genomic data including structure-probing raw reads or processed reactivities, genomic alignments and conservation information, and genomic intervals, e.g., from the CLIP experiments. On the right, a sample selection of the clustering outputs including the overview of the clusters, cluster alignment with LocARNA, RNAalifold consensus structure, and R2R [32] visualization and annotation of the cluster structure by R-scape. Clusters can also be visualized and annotated for the orthology structure conservation predictions.

The GraphClust approach can efficiently cluster thousands of RNA sequences. This is achieved through a workflow with 5 major steps: (i) pre-processing the input sequences; (ii) secondary structure prediction and graph encoding; (iii) fast linear-time clustering; (iv) cluster alignment and refinement, with an accompanying search with alignment models for extra matches; and finally (v) cluster collection, visualization, and annotation. An overview of the workflow is presented in Fig. 1.

More precisely, the pre-processed sequences are individually folded according to the thermodynamic free energy models with the structure prediction tools RNAfold [25] or RNAshapes [26]. A decomposition graph kernel is then used to efficiently compute similarity according to the sequence and structure features of secondary structure graphs. The MinHash technique [27] and inverse indexing are used to identify the initial clusters, which correspond to dense neighborhoods of the graph feature space. Formal description and formulations of kernel and MinHash methods are provided in the supplementary methods. The MinHash clustering approach is very fast with a linear runtime complexity over the number of entries. This accordingly makes GraphClust2 much more efficient than the quadratic all-vs-all approach [19]. It permits the clustering of up to hundreds of thousands of RNA sequences in a reasonable time frame.

After the MinHash clustering step, the initial clusters are refined using the RNA domain-specific tools. First, from the sequences of each initial cluster a UPGMA tree is created to prune the clusters. The pairwise distances of the tree are approximated from LocARNA sequence-structure alignment scores, as is proposed and detailed by Will et al. [17]. This pruning procedure keeps the subtree that has the highest average pairwise alignment on its leaves. Here we use the RNA domain-specific scores from LocARNA alignments, although it would have been possible to compute distances from the generic graph kernel scores. LocARNA scores are used because the runtime complexity is not a concern here, as the pairwise alignments are only computed within each cluster. Each cluster has typically ∼10–100 sequences, which is much smaller than the entire input data. In the second step after pruning, the multiple alignment of each pruned cluster is refined with CMfinder’s expectation maximization algorithm [28]. Third, after the alignment is refined, a homology search using Infernal [21] tools is applied over the entire dataset. For each cluster’s refined alignment, a CM is built using cmbuild. The CM is then used to scan the entire sequence database using cmsearch. This CM homology search step extends the clusters with additional homologs that have been missed in the initial clustering. Finally, the sequences of each cluster are aligned with LocARNA, and the consensus structures are predicted, visualized, and annotated by conservation and covariation metrics.

In an iterative fashion, the steps downstream of the fast clustering can be repeated over the sequences that are not clustered in the previous iteration. GraphClust2 can also compute fuzzy soft overlapping clusters. The option to report overlapping clusters instead of a hard optimal partitioning can be set by the user at the cluster report step. Furthermore, a pre-clustering optional step can be invoked to remove near-identical and redundant sequences using CD-HIT (RRID:SCR_007105) [29]. This pre-clustering would be beneficial for the datasets with high redundancy or very large number of sequences, e.g., metatranscriptomics data.

#### Workflow input

GraphClust2 accepts a set of RNA sequences as input. Sequences longer than a defined length are split and processed with a user-defined sliding window option. Two recommended settings are provided for ncRNA clustering and motif discovery as is discussed in the workflow flavors section. In addition to the standard FASTA formatted input, a collection of auxiliary workflows are implemented to allow the user to start from genomic coordinate intervals in BED format, or genomic alignments from orthologous regions in MAF format, or sequencing data from the SP experiments. Use case scenarios are detailed in the following sections.

#### Workflow output

The core output of the workflow is the set of clustered sequences. Clusters can be chosen either as “hard partitions” having an empty intersection or as overlapping "soft partitions." In the latter case elements can belong to multiple clusters. In-depth information and comprehensive visualizations about the partitions, cluster alignments, and structure conservation metrics are produced (Fig. 1). The consensus secondary structure of the cluster is annotated with base-pairing information such as statistically significant covariations that are computed with R-scape [30]. Evaluation metrics for structure conservation are reported. In the case of MAF input, color-coded UCSC tracks are automatically generated to locate and annotate conserved clusters in the genome browser. The in-browser integrated view of the clusterings makes it possible to quickly inspect the results. The Galaxy server keeps track of the input, intermediate, and final outputs. The clustering results can be shared or downloaded to the client system.

#### Workflow flavors

Two preconfigured flavors of the workflow are offered for the local and the global scenarios, for ease of use without demanding an in-depth knowledge about configuring complex tools. The global flavor aims for clustering RNAs on the whole transcript, such as for annotating ncRNAs of short and medium lengths. The local flavor serves as the motif finder. The motivation has been to deal in an orderly fashion with putative genomic sequence contexts around the structured elements. Prediction methods usually require different settings in these 2 scenarios [31]. The main differences between the flavors are the pre-configured window lengths (∼250 vs ∼100), the aligner parameters, and the hit criteria of the covariance model search (E-value vs “bit score”). The motif finder flavor can be for example used to identify *cis*-regulatory elements, where it is expected to find structured motifs within longer sequences.

As a feature, the fast clustering can be tuned to weigh in sequence-based features. The graph for each entry consists of 2 disjoint parts. The primary part is the structure graph where the vertices are labeled with the nucleotides while the backbone and paired bases are connected by edges. Besides the primary part, a “path graph” can be included to represent the nucleotide string (option -seq-graph-t). By including the path graphs, sequence-only information would independently contribute to the feature vectors.

### Integration of SP data

RNA SP is an emerging experimental technique for determining the RNA pairing states at nucleotide resolution. Chemical treatment with reagents such as SHAPE and DMS [33, 34] provides 1D reactivity information about the accessibility of nucleotides in an RNA molecule. Structure probing (SP) can considerably improve the secondary structure prediction accuracy of RNAs [35–37]. SP-assisted computational prediction methods commonly incorporate the probing data by guiding the prediction algorithms via folding constraints and pseudo-energies [25, 38, 39]. Deigan et al. first introduced the position-specific pseudo-energy terms to incorporate the reactivity information alongside the free energy terms of thermodynamic models [40]. The pseudo-energy term for position *i* is defined as follows:

$$\begin{eqnarray*}
\Delta G_{\text{pseudo-energy}}(i) = m \text{ ln}[1 + \text{reactivity}(i)] + b,
\end{eqnarray*}$$

where parameters *m* and *b* determine a scaled conversion of the reactivities to the energy space. GraphClust2 supports SP data for enabling a guided structure prediction [25, 41]. The SP support is integrated into the pre-processing and the structure prediction steps to generate SP-directed structure graphs.

### Implementation and installation

GraphClust2 is implemented within the Galaxy framework (RRID:SCR_006281) [24]. Galaxy offers several advantages to assist our goal of developing a scalable and user-friendly solution. The platform makes it convenient to deploy complex workflows with interoperable tools. Through the uniform user interface across different tools, it is easier for the users to work with new, unfamiliar tools and freely interchange them. Moreover, the standardized data types will ensure that only inputs with valid types are passed to a tool. Interactive tutorial tours are produced to introduce the user interface and guide the user through sample clustering procedures.

GraphClust2’s toolset has been made publicly available in Galaxy ToolShed [42] and can be easily installed into any Galaxy server instance. GraphClust2 is available also as a standalone container solution for a variety of computing platforms at https://github.com/BackofenLab/GraphClust-2 and can be freely accessed on the European Galaxy server at https://graphclust.usegalaxy.eu.

#### The workflow implementation

GraphClust2 workflow is composed of tools and scripts that are packaged in Bioconda and Biocontainers [43] and integrated into the Galaxy framework. This has enabled automatic installation of the tools in a version-traceable and reproducible way. All functional units and workflows are manually validated and are under extensive continuous integration tests. Strict versioning of tools and requirements ensures reproducible results over multiple different versions of a tool while delivering updates and enhancements.

#### Platform-independent virtualized container

GraphClust2 can be deployed on any Galaxy server instance, simply by installing the GraphClust tools from the Galaxy ToolShed. As a stand-alone solution, a virtualized Galaxy instance based on Linux containers (Docker, rkt) [44] is provided that can be executed on Linux, OSX, and Windows. This largely simplifies the deployment phase, guarantees a reproducible set-up, and makes it instantiable on numerous computation systems from personal computers to Cloud and high-performance computing (HPC) environments. The Docker image is based on the official Galaxy Docker image [45, 46] and is customized to integrate GraphClust2 tools, workflows, and tutorial tours.

### Data

#### Rfam-based simulated SHAPE

A set of Rfam [47] sequences and the associated SHAPE reactivities were extracted from the ProbeAlign benchmark dataset [48]. The simulated SHAPE reactivities have been generated according to the probability distributions that are fitted to experimental SHAPE data by Sükösd et al. methodology [49]. Rfam families containing ≥10 sequences were used. A uniformly sized subset was also extracted, in which exactly 10 random sequences were selected per family to obtain a variation with a uniform unbiased contribution from each family.

#### Arabidopsis thaliana ncRNA DMS-seq

*Arabidopsis* DMS-seq reads were obtained from the SP experiment by Ding et al. [50] (NCBI SRA entries SRX321617 and SRX320218). The reads were mapped to TAIR-10 ncRNA transcripts (Ensembl release-38) [51]. Reactivities were computed for non-ribosomal RNAs based on the normalized reverse transcription stop counts using the Structure-Fold tool in Galaxy [52]. We used Bowtie-2 [53] with the settings recommended by Ding et al. [54] (options –trim5=3, -N=1). Transcripts with poor read coverage tend to bias towards zero-valued reactivities [55]. To mediate this bias, low information content profiles with <1% non-zero reactivities were excluded. To focus on secondary structure predictions of the paralogs that can have high sequence similarity, the graphs were encoded with the primary part without path graphs. Information about the ncRNA families is available in Supplementary Table S4.

#### Orthology sequence extraction from long RNA locus

The genomic coordinates of the longest isoforms were extracted from RefSeq hg38 annotations [56] for FTL mRNA and lncRNAs NEAT1, MALAT1, HOTAIR, and XIST. To obtain the orthologous genomic regions in other species, we extracted the genomic alignment blocks in Multiz alignment format (MAF) [57] for each gene using the UCSC table browser [58] (100way-vertebrate, extracted in August 2018). Alignments were directly transferred to the Galaxy server via the UCSC-to-Galaxy data importer. MAF blocks were concatenated using MAF-Galaxy toolset [59] to obtain 1 sequence per species. An auxiliary workflow for this data extraction procedure is provided. This procedure is notably scalable and can be applied to any locus independent of the annotation availability. Alternatively, the user can provide, e.g., full transcripts or synteny regions [60] for the downstream analysis. For the background shuffled input, Multiperm [61] was used to shuffle the Multiz alignment of the MALAT1 locus.

#### SLBP eCLIP

Binding sites of SLBP were obtained from the ENCODE eCLIP project (experiment ENCSR483NOP) [62]. In the consortium’s workflow, CLIPper [63] is used to extract peak regions of the read coverage data. The peaks are annotated with both p-values and log_2_ fold-change scores. These values are determined from the read counts of the experiment compared with the read counts of a size-matched input. We extracted the peaks with a log_2_ fold-change of ≥4. To diminish the chance of missing the binding motif, the peak regions were extended by 60 nucleotides both up- and downstream. The sequences of the resulting 3,171 binding target regions were used for clustering and motif analysis.

#### Roquin-1 PAR-CLIP

The 16,000+ binding sites of Roquin-1 (RC3H1) were obtained from Murakawa et al. [64] (hg18 coordinates from the associated Max Delbrück Center for Molecular Medicine–Berlin web page). The 5,000 target regions with the highest PAR-CLIP scores were used for the downstream analysis and structural clustering. The binding site sequences were extracted using the extract-genomic-dna tool in Galaxy.

### Structure conservation annotation with Evofold, RNAz, and R-scape

For each of the studied long RNAs, the sequences were extracted from the orthologous genomic regions as detailed in the Data section. Clustering was performed using the motif finder flavor. In the pre-processing step, the sliding window was set to 100-bp length and 70% shift. The LocARNA structural alignments of the predicted clusters were further processed using RNAz [65], Evofold [11], and R-scape [30], to annotate clusters with structure conservation potentials in the generated genomic browser tracks. RNAz uses a support vector machine (SVM) that is trained on structured RNAs and background to evaluate the thermodynamic stability of sequences folded freely versus constrained by the consensus structure. Evofold uses phylo-SCFGs to evaluate a conservation model for local structures against a competing non-structural conservation model. R-scape quantifies the statistical significance of base-pair covariations as evidence of structure conservation, under the null hypothesis that alignment column pairs are evolved independently.

RNAz was invoked (option –locarnate) with the default 50% cut-off for SVM-class probability to annotate the clusters. Evofold was also run with the default parameters over the cluster alignments and supplied with the corresponding hg38-100way UCSC’s phylogenetic tree [57]. Clusters that were predicted by Evofold to contain ≥1 conserved structure with >3 bp were annotated as Evofold hits. R-scape was also applied with the default parameters (i.e., G-test statistics –GTp); clusters with ≥2 significant covariations were annotated. Clusters are constrained to have a depth of ≥50 sequences. Alignments with spurious consensus structure or no conservation were excluded, using a structure conservation index (SCI) filter of 1% [65]. Clusters annotated by ≥1 of the 3 methods are designated as “locally conserved structure candidates.”

### Clustering performance metric

The clustering was benchmarked similarly to our previous work [66], such that the Rfam family to which each input RNA belongs is considered as the truth reference class. The performance is measured using the adjusted Rand index (ARI) [67] clustering quality metric, which is defined as follows

$$\begin{eqnarray*}
\text{Adjusted Rand Index} = \frac{\text{Rand Index} - E(\text{Rand Index})}{1 - E(\text{Rand Index})}.
\end{eqnarray*}$$

The Rand index [68] measures the fraction of the entry pairs that are related in the same way in both the predicted clustering and the reference assignment. *E*[RandIndex] is the expected Rand Index (for extended details please refer to Miladi et al. [66]). The ARI is the corrected-for-chance variation of the Rand index with a maximum value of 1. A better agreement between the predicted clustering and the reference assignment leads to a higher ARI value.

## Results and Discussion

### Clustering performance evaluation

#### Rfam-cliques benchmark

We evaluated GraphClust2 using known RNA families from the Rfam database [47]. The Rfam sequences were obtained from the Rfam-cliques benchmark introduced in our previous work [66]. The Rfam-cliques benchmark contains sets of RNA families at different sequence identity levels and allows for benchmarking a tool for the cases of low and high sequence identities (“Rfam-cliques-low” and “Rfam-cliques-high”). Each variation contains a collection of human members of the Rfam families together with homologs in the other species. Because we wanted to evaluate the performance in a simulated scenario of genome-wide screening, we selected the human paralogs from the benchmarks and measured (using the ARI metric) how well GraphClust1 and the new pipeline GraphClust2 correctly cluster members of the families together.

In comparison to GraphClust1, GraphClust2 provides alternative approaches for the identification of the secondary structures. Using similar configurations as in GraphClust1 [19], i.e., RNAshapes for structure prediction and bit score for CM search hits, the clustering performance of GraphClust2 is similar or better owing to the integration of upgraded tools. However, the alternative configuration of RNAfold for structure prediction and E-value for CM search hits consequently improves the performance (ARI from 0.641 to 0.715 for Rfam-cliques-high; further details in Supplementary Table S1).

#### SHAPE-assisted clustering improves the performance

In the previous benchmark, the clustering relies on the free energy models for secondary structure prediction. A predicted structure sometimes deviates from the real functional structure owing to the cellular context and folding dynamics. In this case, the SP SHAPE data associated with the real functional structure are expected to improve the quality of structure prediction, which in turn should improve the clustering. We wanted to investigate how an improvement in the structure prediction quality at the early clustering steps influences the final clustering results. To draw a conclusion, however, extensive SHAPE data would be needed for a set of labeled homologous ncRNAs, ideally with different sequence identity and under similar experimental settings. Currently, such collection of data, especially over multiple organisms, is still unavailable. However, because the SP is turning into a standard and common procedure, data of such nature are expected to become available soon.

**Figure 2:**
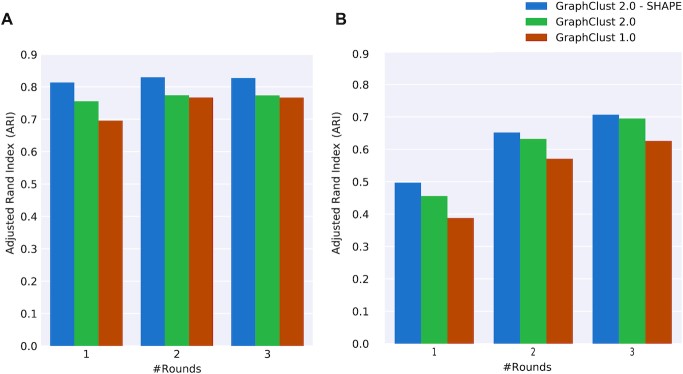
Clustering quality performance over Rfam-based ProbeAlign benchmark dataset and the associated simulated SHAPE data. For comparison, GraphClust2 and GraphClust1 performances are also shown. Incorporating the simulated SHAPE data assists in the clustering performance. (A) ARI clustering quality metric for 1–3 rounds of iterative clustering. ARI of the clusterings did not improve noticeably after 3 rounds. (B) Similar to (A) but for uniformly sized families, such that precisely 10 sequences are randomly extracted per family.

One solution to the mentioned data scarcity is provided in the literature [49], by simulating the experimental generation of a SHAPE profile from the real functional structure. Here, starting from a set of manually curated reference structures, the idea is to simulate SHAPE profiles that reflect the known reference structures. We used the benchmark from ProbeAlign [48] (see Materials and Methods for details). Fig. 2 shows the effect of incorporating simulated SHAPE data on clustering by guiding the structure prediction. As can be seen, the incorporation of SHAPE data has improved the clustering performance. Notably, an improvement can be achieved in fewer rounds of clustering iterations.

### GraphClust2 is scalable

To validate GraphClust2’s scalability and linearity claims, we used a millions-sequence biological dataset. GraphClust2 is implemented with comprehensive support for parallel computation using the Galaxy framework. The MinHash-based clustering step is the only step in which the entities are evaluated altogether to identify the dense neighborhoods as clusters. Thanks to the MinHash technique, this step has only a linear complexity (see Materials and Methods and Supplementary Section S1). To empirically validate this, we clustered a large metatranscriptomic dataset of a marine sample from Pfreundt et al. [69]. After merging the paired-end reads, the metatranscriptome contained 3,594,198 sequences with an average length of 250 bp. To filter highly similar sequences, we performed sequence-based pre-clustering with CD-HIT set at a 90% similarity threshold. This produced ∼913,000 sequences with a total of 195 million bases. GraphClust2 identified several large clusters of sizes >100 in 1 round. Translation-complex–related RNAs (transfer RNA, large subunit, and 5s ribosomal RNAs) were among the dominating ncRNA classes, matching the expectation due to the high expression levels of the families (see Supplementary Table S2 for further details). Clustering the entire 3.6 million sequences took <1 day on the European Galaxy server. To check the runtime growth over number of inputs, we measured the wall clock runtime for sub-samples of various sizes on the European Galaxy server. GraphClust2 robustly scaled with a linear trend over the size of the input (Supplementary Fig. S1).

### Clustering *Arabidopsis* ncRNAs with DMS-seq *in vivo* SP data

As shown in the previous section, we expect SP information to improve the clustering. Information about the structure formations *in vivo* can be obtained from SP techniques by determining the nucleotide-resolution base reactivities, where positions with high reactivity indicate unbound bases. Recently, high-throughput sequencing has enabled SP to be applied in a genome-wide manner, thus providing SP reactivities of an entire transcriptome [70]. In this way, a large amount of SP data can be obtained. Despite the availability of genomic-wide SP data, its application for transcriptome-wide structure analysis is promising [71] but has remained largely underutilized.

#### *Enhanced ncRNA annotation with*in vivo*SP data*

**Figure 3:**
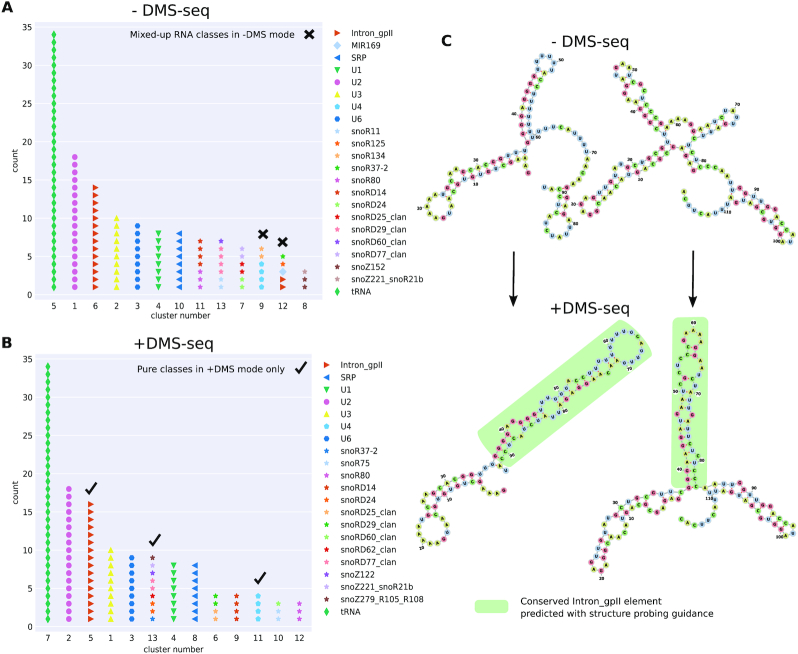
Clustering ncRNAs from *Arabidopsis thaliana* with and without incorporating *in vivo* DMS-seq SP data [50]. (A) The predicted clusters without probing data (−DMS-seq) are depicted and the reference family labels are superimposed. Clusters 9 and 12 contain mixed RNA classes. Here, the Group II introns RNA family is split between clusters 6 and 12. (B) Similar to A, for +DMS-seq where experimental SP data have been used to guide the structure prediction of the generated graphs with pseudo-energy terms. In +DMS-seq mode, only clusters with members from single RNA classes are produced. (C) We inspected the predicted structure in more detail. The 2 transcripts of the Intron_gpII family are shown that exhibit substantial structure deviations between their minimum free energy structures (−DMS-seq) and the structures guided by the probing data (+DMS-seq). The structures are predicted using Vienna RNAfold and drawn with forna [76]. The green highlighted branches correspond to the conserved reference structures from Rfam that are correctly predicted only when the DMS-seq reactivities are incorporated.

We thus evaluated how the task of clustering and annotation of ncRNAs can benefit from such genome-wide probing experiments. For this, we compared clusterings of *A. thaliana* ncRNAs with and without considering the DMS-seq data by Ding et al. [50] (see Materials and Methods). Owing to the relatively high sequence similarity of the annotated paralogous ncRNAs of *A. thaliana*, the ARI is high even when no SP data are considered (−DMS-seq mode ARI 0.88). Nonetheless, the quality metric is slightly improved by incorporating the SP data (+DMS-seq mode ARI 0.91). We further manually inspected the quality of the produced clusters. Fig. 3 shows the enhanced results for identifying ncRNA classes by using GraphClust2 with *in vivo* probing data. In the +DMS-seq mode (Fig. 3B), all detected clusters are pure RNA classes, while the −DMS-seq mode (Fig. 3A) produces mixed-up clusters for the Group II Introns family plus small nucleolar RNA, microRNA, and U–small nuclear RNA classes. For example, as seen in Fig. 3C, the SP data improve the structure prediction by predicting a conserved stem for 2 of the Group II Introns only in the +DMS-seq mode, which leads to 1 pure cluster for the family.

### Discovering locally conserved structure in long RNAs

RNA-sequencing experiments from biological conditions often result in differentially expressed transcripts, which are studied for functionality and regulatory features. A differential expression hints at putative regulatory effects. An orthogonal source of information for the functional importance of a transcript is phylogenetic conservation patterns. For long non-coding RNAs, however, sequence conservation is usually low, imposing limitations on the sequence-level conservation analysis. This fact has been one motivating reason for a collection of recent studies to explore the conservation and functionality of lncRNAs at the secondary structure level [72–74]. A majority of the studies have been focusing on identifying widely spanned structures, postulating the existence of a to-be-discovered single global structure. However, some of the reported conservations have been challenged for lacking trustworthy base-pair covariations in the alignments [30].

Looking for locally conserved secondary structures in lncRNAs is alluring for several reasons. First, with an increase in the base pair span length the prediction quality decreases [31], which implies that global structure prediction for long RNAs tends to be inaccurate. Second, the structure of a transcribed RNA structure is influenced by RNA-binding proteins *in vivo*, and thus a predicted global structure likely deviates from the real functional structure. Third, in many cases and similar to the untranslated regions (UTRs) in mRNAs, only a locally conserved structural motif is expected to suffice to perform a function, independent of the precise global structure. We thus revert to a frequently used strategy in the RNA field, namely, to look for locally conserved structural motifs. We wanted to evaluate whether we can use GraphClust2 for this purpose.

It should be noted that distinguishing conserved structures from background genomic sequence similarity using base-pair conservation signals is a challenging task. Genome-wide screening studies over genomic alignments require adjusted thresholds for statistical significance discovery and report up to 22% [4] false discovery rates, which can be even higher [75]. Despite this and owing to the persistent expansion of genomic data, the depth and quality of genomic alignments are continually increasing. Currently, there is a lack of off-the-shelf tools for comprehensively analyzing locally conserved structural elements of a specific locus. Here based on GraphClust2, we propose a data extraction and structure conservation detection methodology (as detailed in the Materials and Methods) that can readily be used for desired loci and genomic alignments to identify “candidates” with locally conserved structure potentials.

An advantage of this clustering approach over traditional screening methods is its ability as an unsupervised learning method, for not imposing explicit presumption on the depth or number of predicted motifs. This also makes it possible to find the locally conserved structures in the regions where a subset of species do not have a conserved structure. Furthermore, this approach does not require a precise co-location of the conserved elements within the transcript, in contrast to traditional alignment-based screening approaches. A further advantage is the availability of the solution in the Galaxy framework because it provides a rich collection of assets for interactive data collection and analysis of genomic data. We used the 100way vertebrate alignments to extract the orthologous genomic regions for each of the studied RNAs in human and other vertebrates. Each of the orthologous sequences is split into windows, which are then clustered by GraphClust2. The alignment of each cluster has been further annotated with some of the best-practice complementary methods in assessing covariation patterns and structure conservation potentials, namely, RNAz, Evofold, and R-scape (see Materials and Methods for details). In the following section, some example studies are presented.

### Locally conserved candidates with reliable alignments are observable but uncommon

**Figure 4:**
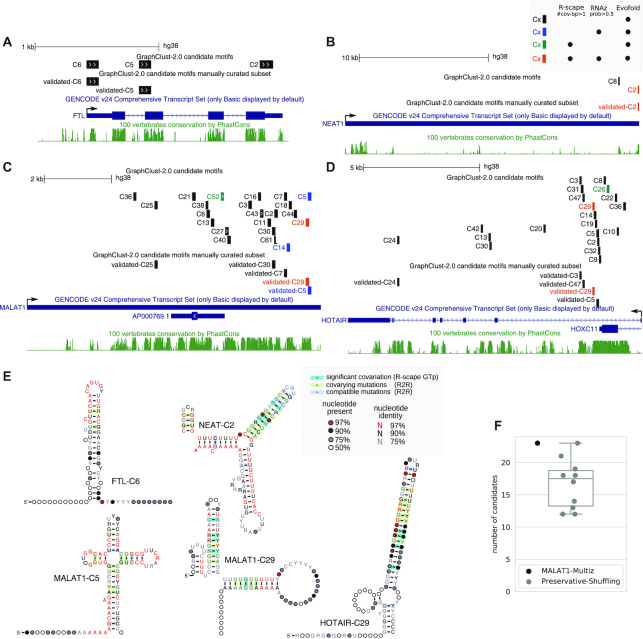
Locally conserved structured elements predicted in FTL mRNA and lncRNAs NEAT1, MALAT1, and HOTAIR. (A–D) Locations of the predicted clusters relative to the transcript on the human genome. The clusters under the manually curated subset track, labeled as validated, have passed a qualitative manual screening to exclude unreliable structural alignments (see Results and Discussion). (E) Consensus secondary structure for some of the clusters with reliable sequence-structure alignments. Secondary structures are visualized with R2R [32]; statistically significant covariation is computed by R-scape and manually overlaid on the R2R visualizations. The alignments are visualized in Supplementary Figs S6–S11. (F) Comparison between the number of predicted candidate motifs of MALAT1 vs 10 times Multiperm’s preservative shufflings of the same genomic alignment.

We investigated clustering of orthologous genomic regions of FTL mRNA and 4 well-studied lncRNAs, using the approach described before. The selected lncRNAs have been previously reported for having loss-of-function phenotypes [77, 78]. In Figs 4A–D and Supplementary Fig. S3 the locations of locally conserved candidates are displayed. These locations are automatically generated by GraphClust2 from clusters with conserved structures (“candidate motifs” track). The track is automatically annotated and filtered using the computed metrics of the Evofold, RNAz, and R-scape tools (see Materials and Methods and Fig. 4B legend). For these studied lncRNAs, an additional track (“manually curated subset”) is provided. The track is the selection subset of the candidate motifs track that are manually further screened and selected by stringent expert criteria. The intention was to identify confident conserved elements that can be used, e.g., for mutational experiments. The clusters were manually curated in a qualitative manner by inspecting the alignments, their consensus structures as well as the conservation metrics. Only the highly reliable structural alignments that posed a good level of covariation and were not deemed to be alignment artifacts were selected. The main filtering out criteria were as follows: singleton compensatory mutations, avoidable column shifts producing artificial mutations, absence of any region with a basic level of sequence conservation, and similar frequencies of variations in both unpaired and compensatory mutated paired regions. Below we describe the observations from these lncRNAs’ conservation analyses.

#### FTL

The *cis*-acting iron response element (IRE) is a conserved structured element that is located on the 5′ UTR of *FTL* (ferritin light chain) and several other genes. Mutations that disrupt the hairpin structure of IRE cause disease phenotypes by changing the binding affinity of a regulatory iron response protein [79, 80]. As a proof of concept, we applied GraphClust2 to discover structural motifs in the homologous regions of the *FTL* mRNA. The IRE element was identified as 1 of the 3 clusters detected by Evofold (Fig. 4A).

#### NEAT1

The NEAT1 analysis suggests very limited but also very reliable structure conservation at the 3′ end of the transcript that is consensually detected by the 3 evaluated tools.

#### MALAT1

MALAT1 has a relatively higher level of sequence conservation among the 4 studied lncRNAs. A higher number of clusters were predicted with a couple of reliable candidates that lean towards the 3′ side of the transcript.

To examine how many of the detected motifs are expected to be false-positive predictions, we ran the pipeline on 10 shufflings of the MALAT1 100way alignment. For the shuffled background, we used Multiperm to preserve the gap structure, local conservation structure patterns, and the relative dinucleotide frequencies of the MALAT1 alignment [61]. On average 16.7 candidates were reported for the shuffled genomic alignments, in comparison to the 23 candidates reported for the genomic alignment (Fig. 4F). In the predicted set of candidates from the clustering of the shuffled background alignments, none was consensually annotated by the 3 methods. For the applied alignment depths and thresholds, Evofold had a considerably higher discovery rate than R-scape and RNAz. In total out of 10 shuffles 167, 9, and 0 clusters were predicted to have a conserved structure by Evofold, R-scape, and RNAz, respectively.

#### HOTAIR

The predicted candidates for HOTAIR are all located on the intronic regions of the precursor lncRNA. Clustering from the second exon, through skipping the first exon and intron, did not change this observation. A dense number of candidates can be noticed on the first intron that is overlapping with the promoter region of HOXC11 on the opposite strand. Most notably is the candidate cluster HOTAIR-C29, which is highly enriched in G-U wobble base pairs (Fig. 4E). In contrast to Watson-Crick GC and AU base pairings, the GU reverse complement AC is not a canonical base pair [81]. Therefore, this structure can only be formed on the antisense RNA and not on HOXC11’s sense strand.

#### XIST

The XIST candidates are mainly located on the repeat regions and are paralog-like (Supplementary Fig. S3). Manual evaluation of the cluster structural alignments was inconclusive. In the mixture of paralog-like and homolog-like sequences of the cluster alignments, it was not possible to conclude whether the structural variations are merely artifacts of sequence repetition or compensatory mutations of hypothetical structure conservation.

### Clustering RNA binding protein target sites

#### SLBP eCLIP

**Figure 5:**
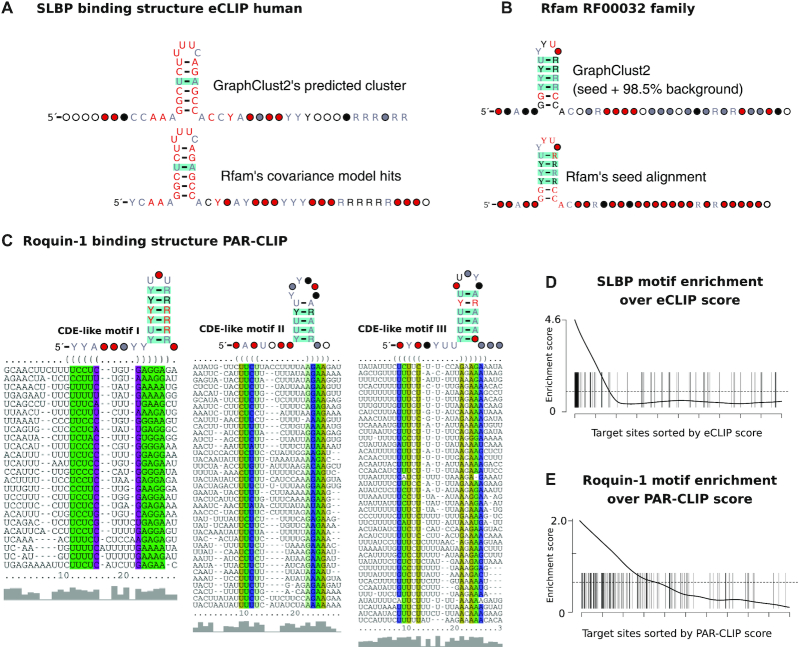
Structured RNA motifs identified by clustering SLBP and Roquin-1 public CLIP data with GraphClust2. (A) The consensus secondary structure of the predicted human SLBP motif from eCLIP data versus the consensus structure of cmsearch hits from Rfam’s CM for histone 3′ UTR stem-loop family RF00032. (B) Top, The consensus secondary structure of the predicted structure motif from clustering 3,000 sequences composed of RF00032’s 46 seed sequences and 2,056 background shuffled full sequences as 98.5% background noise. (B) Bottom, Rfam’s reference structure for RF00032 seed alignment. (C) The consensus secondary structures and alignments of the 3 clusters with defined consensus structures. The 3 motifs overlap and have varying loop sizes and uridine content. The structures are akin to the previously validated constitutive decay element (CDE) in TNF-α that is a target of Roquin-1. (D, E) Gene set enrichment plot of SLBP and Roquin-1 motifs according to the corresponding CLIP scores. SLBP eCLIP has a high enrichment of the stem-loop with strong density in the first 100 target sites. Roquin-1 PAR-CLIP data have a lower enrichment score and low presence in the top 100 target sites. The difference in the enrichment is likely due to the specificity of Roquin-1 that has multiple RNA binding domains and false-positive biases of the PAR-CLIP protocol. Scalable clustering assists in overcoming these biases to identify the CDE-like elements. Structures are visualized by R-scape; the color for significant base-pair covariations matches the legend in Fig. 4E. Enrichments are plotted with the Limma R package [88].

A well-characterized example of an RBP with specific structural preferences is SLBP (histone stem-loop–binding protein). We clustered target sites of human SLBP using the publicly available eCLIP data [62]. The largest cluster with a defined consensus structure bears statistically significant base-pair covariations. The structure matches the SLBP’s Rfam family “histone 3′ UTR stem-loop” (RF00032). Using the family CM to identify SLBPs on the eCLIP data, we were able to predict exactly the same stem-loop structure with the same level of base-pair covariation (Fig. 5A). GraphClust2 and Rfam’s CM hits have >95% overlap. These correspondences demonstrate that GraphClust2 can identify the consensus structure element from CLIP data with a high sensitivity.

The stem-loop structure of the eCLIP data has a lower covariation level than Rfam’s seed alignment (Fig 5A vs B). This is because the Rfam data are phylogenetically diverse (RF00032 seed: 28 species) while eCLIP data exclusively have a human origin (eCLIP: K562 cell line). We checked how GraphClust2 would perform if the eCLIP data from diverse organisms were available. To simulate eCLIP data with high covariation level, we mixed up the 46 seed sequences of the RF00032 family with 2,954 shuffled sequences to obtain 3,000 sequences such that SLBP is convoluted with 98.5% background. The sequences from the full RF00032 set were shuffled to obtain a background of the same length and nucleotide content distribution. As can be seen in Fig. 5B, GraphClust2 successfully managed to cluster the family entries as 1 cluster. Here, the cluster has the same stem-loop in the consensus secondary structure with the same covariation level as Rfam’s reference structure.

#### Scalable clustering identifies novel CDE-like elements in Roquin-1 PAR-CLIP data

Roquin-1 is a protein with conserved double-stranded RNA binding domains that binds to a constitutive decay element (CDE) in TNF-α 3′ UTR and several other mRNAs [82, 83]. Roquin-1 promotes mRNA decay and plays an essential role in the post-transcriptional regulation of the immune system [84]. We clustered the binding sites of the publicly available Roquin-1 PAR-CLIP data [64] with GraphClust2. Clustering identified structured elements in 3 dominant clusters with defined consensus structures. Fig. 5C shows the alignments and consensus structures of the 3 clusters. The consensus structures are similar to the previously reported CDE and CDE-like elements [85].

It should be noted that the union of Roquin-1’s CDE-like motifs have a lower enrichment score based on the PAR-CLIP ranks, in comparison to the SLBP motif based on the eCLIP ranks (Figs 5D and E and Supplementary Fig. S4). For example, only 6 of the CDE-like motifs are within the top 100 PAR-CLIP binding sites. Therefore, only the clustering of a broader set of binding targets, with a permissive score threshold, allows the CDE-like elements to be reliably identified. We hypothesize that 2 reasons contribute to the observed distinction. First, eCLIP is an improved protocol with a size-matched input to capture background RNAs of the CLIP protocol [62]. On the other hand, PAR-CLIP is known to have relatively higher false-positive rates [86]. Second, the ROQ domain of Roquin-1 has 2 RNA binding sites, 1 that specifically recognizes CDE-like stem-loops and 1 that binds to double-stranded RNAs [85, 87]. This would likely broaden the Roquin-1 binding specificity beyond CDE-like stem-loops.

#### BCOR 3′ UTR is a prominent conserved target of Roquin-1

**Figure 6:**
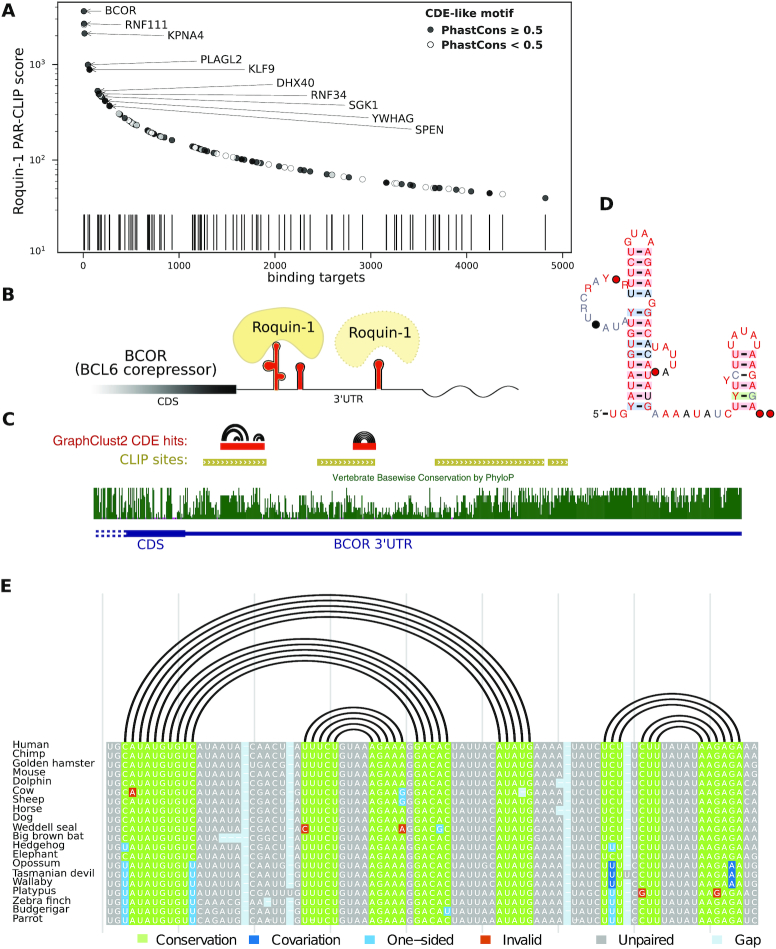
(A) The distribution of the Roquin-1 target sites bearing the CDE-like motifs on the 3′ UTRs of genes, according to the binding affinity scores. Top 10 highest binding sites with a conserved CDE-like motif are labeled with the associated gene names. (B) Roquin-1 binds to a highly conserved double stem-loop element on the 3′ UTR of BCOR (BCL6 CoRepressor) with very high affinity. Another CDE-like element with lower affinity downstream of the first element is also spotted. CDS: coding sequence. (C) Conservation track of BCOR 3′ UTR end plus the location of the CDE-like motifs on the negative strand of the locus on the human X chromosome. (D) R2R visualization for the RNAalifold consensus secondary structure of the conserved double stem-loop element from the vertebrate Multiz alignment. (E) Genomic alignment of the double stem-loop for 20 selected species annotated with the consensus structure and the base-pair covariation information. Alignments and compensatory mutations are visualized with R-chie package [91]. The alignment overview for all the available species as extracted from 100way Multiz is provided in Supplementary Fig. S12.

We performed a follow-up conservation study over the identified CDE-like motifs from the clustering of Roquin-1 binding sites (Fig. 6A). By investigating RNAalifold consensus structure predictions for Multiz alignments of the top 10 binding sites of the conserved candidates, the BCOR’s CDE-like motif was observed to have a highly reliable consensus structure with supporting levels of compensatory mutations. Interestingly the reported CLIP binding site region contains 2 conserved stem-loops . Downstream of this site, further binding sites with lower affinities can be seen, where one site contains another CDE-like motif. So in total BCOR’s 3′ UTR contains 3 CDE-like motifs (Fig. 6B and C). The shorter stem-loop, at the upstream binding site, has a double-sided base-pair covariation and the longer stem-loop contains bulges and compensatory 1-sided mutations (Fig. 6D and E). BCOR has been shown to be a corepressor of BCL6, which is a major sequence-specific transcription repressor. BCL6 expression is tightly regulated and induced by cytokine signaling such as interleukins IL4, IL7, and 21 [89, 90]. Overall these results propose BCOR to be a functionally important target of Roquin-1 and assert the role of Roquin-1 in regulating follicular helper T cell differentiation and immune homeostasis pathways [83].

## Conclusion

We have presented a method for structural clustering of RNA sequences with a web-based interface within the Galaxy framework. The linear-time alignment-free methodology of GraphClust2, accompanied by cluster refinement and extension using RNA comparative methods and SP data, were shown to improve the detection of ncRNA families and structurally conserved elements. We have demonstrated on real-life and complex application scenarios that GraphClust2 provides an accessible and scalable way to perform RNA structure analysis and discovery.

GraphClust2 provides an integrative solution, which can start from raw high-throughput sequencing and genomic data and ends with predicted motifs with extensive visualizations and evaluation metrics. The users can benefit from the vast variety of the bioinformatics tools integrated by the Galaxy community and extend these applications in various ways. Thus, it will be for the first time possible to start from putative ncRNAs in transcriptomic RNA-sequencing studies and immediately cluster the identified transcripts for annotation purposes in a coherent manner.

## Availability of Source Code and Requirements

- Project name: GraphClust2
- Project repository: https://github.com/BackofenLab/GraphClust-2
- Project home page: https://graphclust.usegalaxy.eu
- Galaxy tools repository: https://github.com/bgruening/galaxytools/tree/master/tools/GraphClust
- Operating system(s): Unix (Platform independent with Docker)
- GraphClust2 Docker image: https://hub.docker.com/r/backofenlab/docker-galaxy-graphclust
- License: GNU GPL-v3
- RRID:SCR_017286

## Availability of Supporting Data and Materials

The data presented here that illustrate our work are available from Zenodo [92], and all steps taken for data analysis are accessible via a collection of Galaxy histories from the project home page at the European Galaxy server (https://graphclust.usegalaxy.eu). Archival copies of the GitHub repositories are also available from the GigaScience GigaDB repository [93].

## Additional Files

**Supplementary information**: Supplementary Methods and Results are available via the additional file associated with this article.

Supplementary Figure S1: Clustering scalibility and runtime evaluation using the marine metatranscriptomic dataset.

Supplementary Figure S2: Characteristics of the clusters predicted from the long RNA conservation analysis.

Supplementary Figure S3: Locally conserved structured elements predicted in XIST lncRNA.

Supplementary Figure S4: Distribution of SLBP motifs over the eCLIP scores.

Supplementary Figure S5: Color legend for Supplementary Figures S6-S11.

Supplementary Figures S6-11: Sequence-structural alignments of the selected clusters from Figure 4. Supplementary Figure S12: Overview of BCOR's CDE-like alignment for all the available species.

Supplementary Table S1: Clustering benchmark performance using Rfam-cliques datasets.

Supplementary Table S2: Statistics of the clusters predicted from the marine metatranscriptomic study.

Supplementary Table S3: Clustering runtimes of the long RNA conservation and CLIP analyses.

Tabular file T1: The genomic coordinates, structure conservation scores and statistics of the GraphClust2 candidates in the long RNA conservation analysis.

Tabular file T2: Genomic coordinates, gene names and conservation information of the identified CDE-like motifs from Roquin-1 CLIP data.

## Abbreviations

ARI: adjusted Rand index; bp: base pairs; CDE: constitutive decay element; CLIP: cross-linking immunoprecipitation; CM: covariance model; DMS: dimethyl sulfate; HPC: high-performance computing; lncRNA: long non-coding RNA; MAF: Multiz alignment format; mRNA: messenger RNA; NCBI: National Center for Biotechnology Information; ncRNA: non-coding RNA; RBP: RNA binding protein; SCFG: stochastic context-free grammar; SHAPE: selective 2′-hydroxyl acylation analyzed by primer extension; SP: structure probing; SRA: Sequence Read Archive; UCSC: University of California Santa Cruz; UTR: untranslated region.

## Funding

This work was supported by German Research Foundation Collaborative Research Centre 992 Medical Epigenetics (DFG grant SFB 992/1 2012) and German Research Foundation (DFG grants BA 2168/13-1 and BA 2168/14-1). The article processing charge was funded by the German Research Foundation (DFG) and the Albert Ludwigs University Freiburg in the funding programme Open Access Publishing. R.B. is funded by the Deutsche Forschungsgemeinschaft (DFG, German Research Foundation) under Germany´s Excellence Strategy - EXC-2189 - Project ID: 390939984. Gefördert durch die Deutsche Forschungsgemeinschaft (DFG) im Rahmen der Exzellenzstrategie des Bundes und der Länder - EXC-2189 - Projektnummer 390939984.

## Competing Interests

The authors declare that they have no competing interests.

## Acknowledgments

We thank Freiburg Galaxy team for their support. We thank Sean Eddy for the helpful comments and discussions. We also thank Sita J. Saunders and Mehmet Tekman for providing feedback about this manuscript.

## Supplementary Material

giz150_GIGA-D-19-00089_Original_Submission

giz150_GIGA-D-19-00089_Revision_1

giz150_GIGA-D-19-00089_Revision_2

giz150_GIGA-D-19-00089_Revision_3

giz150_Response_to_Reviewer_Comments_Original_Submission

giz150_Response_to_Reviewer_Comments_Revision_1

giz150_Response_to_Reviewer_Comments_Revision_2

giz150_Reviewer_1_Report_Original_Submission Fabrizio Ferre -- 5/16/2019 Reviewed

giz150_Reviewer_2_Report_Original_Submission Ioanna Kalvari -- 5/27/2019 Reviewed

giz150_Reviewer_2_Report_Revision_1 Ioanna Kalvari -- 10/14/2019 Reviewed

giz150_Supplemental_Files
