# Supplementary material for: GraphClust2: Annotation and discovery of structured RNAs with scalable and accessible integrative clustering
Source: Gigascience. 2019 Dec 6;8(12):giz150. doi: 10.1093/gigascience/giz150 (PMC6897289; doi:10.1093/gigascience/giz150)
Supplement: giz150_Response_to_Reviewer_Comments_Original_Submission [file giz150_response_to_reviewer_comments_original_submission.pdf]

Author's Response To Reviewer Comments

Close

First, we would like to thank the reviewers and the editors for their encouraging and constructive comments on our manuscript. We have extended the manuscript, clarified the methodologies and performed additionally new experiments. Below we provide the point-to-point responses with referring to the corresponding modifications of the manuscript and the updated and extended results. To facilitate tracking the changes, paragraphs with a major amount of change are highlighted in blue within the manuscript.

Reviewer #1:
> In the presented manuscript, the authors describe a tool for the clustering of RNAs based on secondary structure similarities. Their approach can find application in the classification of RNAs and in finding structural motifs. The method has stand-alone implementations as well as it is integrated within the Galaxy framework, with the aim of facilitating and standardizing its usage. While the manuscript is globally well written, some aspects of the method could be better clarified. The authors might want to consider the following points:

> 1. The clustering algorithm description is confusing. First, a graph kernel is used to identify RNAs forming initial clusters, which are then refined using UPGMA and CMfinder, and finally a covariance model is built for each cluster and used to scan the remaining RNAs. I don't understand how UPGMA and CMfinder are employed.

#Authors:
We thank the reviewer for their valuable comments. In this revision, the clustering description in "Methods overview" has been extensively updated and supplied with extended descriptions for better clarity. Inline below comes answers to the reviewer's question which are now mirrored in the manuscript as well.

> Are they alternative to each other, or integrated in some undocumented way?

#Authors:
They are both used in our pipeline. First, a UPGMA is applied to each of the MinHash clusters to prune the set of sequences, then CMfinder is followed up on each of the pruned sets.

> Which information provided by UPGMA and/or CMfinder is used to compute the covariance model?

#Authors:
The UPGMA step removes potential outliers within each cluster, which deemed similar according to the Minhash-Graph-kernel features but are later detected as dissimilar at the RNA-specific structural level according to the LocARNA scores. Afterward, the CMfinder level refines the structural alignment to improve identifying local structures.

> Moreover, the iterative nature of the clustering algorithm is not evident from their description in the Materials and Methods section. I only realized that by looking at Fig. 2. I guess that the initial covariance models are progressively recomputed by adding new RNAs but, if it that's the case, a more detailed description of the procedure must be provided;

#Authors:
Thanks for this comment, we improved the corresponding section and made it more clear. The iteration step description is extended and moved to the beginning of the next paragraph, in the "Methods overview" (after cluster collection, before pre-clustering).
The sequences, which are not assigned to a cluster until this round, are compared in the fast clustering

step to identify new dense centers in the feature space. Eventually, new covariance models are generated and used to find further hits for the cluster.

> 2. Is the LocARNA score an ultrametric? Can the graph kernel similarity scores be converted into a distance to feed UPGMA?

#Authors:
For filtering the outliers within each cluster, we follow and use the strategy of the LocARNA tool, which is detailed in Will et al. work [Plos Comp. Bio. 2007. doi:10.1371/journal.pcbi.0030065]. The distance is approximated from the pairwise alignment score by LocARNA package. The UPGMA step is merely used to prune outlier sequences within each cluster and not for predicting the clusters. So the UPGMA and the distance approximation does not have a major effect on the clustering.
The kernel scores can be used to produce the distances with a lower runtime. However, here we use the LocARNA score since it is domain-specific and designed for structured RNAs. Because the quadratic pairwise comparison is only for the sequences within each cluster (usually ~10-100 sequences), the runtime is not a concern. We have extended the relevant description within the manuscript.

> 3. From the "Workflow output" section in M&M, it seems that fuzzy clusters (called soft clusters in the manuscript) can be obtained, but it is not explained how;

#Authors:
Thanks for the comment. An explanation is added to the "Methods overview" about the treatment of fuzzy/soft clusters.
A sequence can potentially match to multiple CMs. This would produce fuzzy/soft clusters, two clusters with overlap member ratio above the threshold are merged in the cluster collection step. A user-definable option to perform soft clustering is provided in the collection step.

4. In the Results and Discussion, section "Locally conserved candidates…", manual checking and filtering is reported. I wonder which is the impact of these expert manual screens, and which results a non-expert could expect to obtain;

#Authors:
In Figure 4. two tracks are reported. The first one, "candidate motifs", is the GraphClust2 automatically generated predictions and the second one, "manually curated subset", has been selected by screening candidates of the first track. So the non-expert gets the "candidate motifs" as a result which are supplemented with annotation scores from the three methods (RNAz, EvoFold2, R-scape). We have updated the text to clarify the difference.

> 5. It is not clear how RNAz, Evofold and R-scape are used, whether they provide filtering criteria or are just used to annotate and describe the results;

#Authors:
The three tools are invoked after clusters are predicted and collected. They are used to evaluate the structure conservation signals and identify highly conserved structural elements.
To clarify comments 4 & 5, we have revised the filtering section in the manuscript and added a specific reference to the M&M section.

> 6. Could running times for the described examples be provided?

#Authors:
We have added the runtime of the experiments as a supplementary Table S2. Furthermore, to demonstrate and verify GraphClust2's scalability, we have clustered a large metatranscriptome dataset. Please see the second results section about the runtime analysis. Besides, we have provided a new supplementary Figure S1.

Reviewer #2:
> The clustering of ncRNAs is an add-on to existing technologies of ncRNA annotation. This is done by allowing de-novo identification of ncRNA families and motifs, compared with the literature based family building process, starting from known ncRNA sequences which work as SEEDs. Also, tools that cluster

ncRNA sequences are scarce and, in most cases, publicly unavailable, therefore projects such as this are essential. Building a ncRNA family requires a number of repetitive curation steps aiming to improve the initial multiple sequence alignment and consensus secondary structure. For that reason, tools that omit the expert contribution require thorough assessment.

#Authors:
We sincerely thank the reviewer for their thoughtful comments and inspections of our submission. GraphClust2 tool is designed to complement available procedures and assist both non-experts as well as experts in speeding up the process of identifying and annotating ncRNAs.

> The authors nicely demonstrated that the inclusion of structure probing data such as SHAPE, can improve the clustering performance of GraphClust2 when compared with its preceding version, namely GraphClust. However, the results of the experiment based on eCLIP data left me questioning the quality of the clustering methodology and the test dataset.

#Authors:
We again thank the reviewer for their careful inspection. We have included new experiments and extended the eCLIP panels in Figure 5, which are detailed in our responses below. We believe that these enhancements would have resolved the false impression of performance, which was caused by having structures of two inherently different data sources (Rfam vs. eCLIP) side-by-side.

> The secondary structure generated from the largest cluster from the eCLIP data experiment, shows loss in base-pair covariation compared with the consensus secondary structure obtained from Rfam. It is very important to ensure the clustering works efficiently enough, as base-pair covariation is evidence that the secondary structure of a family of ncRNAs is correct.

#Authors:
The authors cannot agree more with the reviewer that the bp-covariation is strong evidence of structure-level conservation. For this reason, we have provided and highlighted the importance of base-pair covariations via R2R-Rscape plots, annotated structural alignments, conservation/covariation scores, which we have included in several plots. Following the reviewer's advice, we have performed additional experiments to validate the GraphClust2 performance and resolve the SLBP's covariation concern. Our response points are summarized here and the point-by-point answers come further below.

- The eCLIP data is not similar to the Rfam seed data. Therefore the structures are not expected to look the same. The eCLIP data is from a single human cell line and ortholog-only. This is contrary to the more diverse Rfam's seed data, which originates from 28 organisms and multiple experiments.

- The base-pair covariation of the applied human eCLIP data is inherently much less than Rfam.

- In line with the reviewer's suggestion, we have now validated the GraphClust2 performance for the eCLIP data by counterpart comparison with the Rfam's family covariance model. Rfam's CM is the ideal golden model since it has been built using the highly diverse set and covarying structures of Rfam's seed alignment. The Rfam's CM cmsearch hits are almost the same as (~96% overlap) the GraphClust2 prediction. Both GraphClust2 and Rfam showed the same (low) level of covariation (new Fig. 5-A).

- Following the reviewer's suggested experiment, we used GraphClust2 to cluster Rfam's seed sequences that were mixed up with 98.5% noise. GraphClust2 predicted and constructed the secondary structure with the same high level of covariation as the Rfam reference structure (new Fig. 5-B).

- The performance has been quantitatively measured and transparently demonstrated using the independently-designed Rfam-cliques and ProbeAlign dataset (Fig. 2 and Table S1). Those datasets have multiple levels of sequence identity and designed to convey high covariation.

> The following points could help investigate this further:
> 1. How taxonomically diverse is the dataset used? Although the dataset apart from human sequences also includes sequences from other species - which the authors do not mention in the manuscript - is likely not diverse enough. Histone3 family (RF00032) is built from 46 sequences coming from 28 distinct species

#Authors:
Following the reviewer's question, we have carefully analyzed the eCLIP and Rfam family datasets. The eCLIP data is human-only and cell-type-specific. From the eCLIP paper : "We generated 102 eCLIP experiments for 73 diverse RBPs in HepG2 and K562 cells" [(Van Nostrand et al. 2016)]. Therefore we have extracted regions from the human genome.
So it does not include other species and is therefore not comparable to the Rfam's RF00032 seed dataset. We have clarified this as well in the section "SLBP eCLIP" paragraph under Materials&Methods-Data.

> 2. What is the sequence identity threshold and how was it decided for the best clustering results? This is something the authors did not mention in the manuscript and testing different thresholds could potentially result in gain of base-pair covariation support

#Authors:
GraphClust2 has no explicit sequence identity threshold setting. The clustering procedure uses Graph Kernel for comparing secondary structure graphs, so there is no explicit definition of the sequence identity for the cluster identification. This procedure is akin to counting the matching-kmers of two sequences but in the graph 2D space. Also, in the cluster extension step, we use the CM bitscore/E-value for the hit thresholds.

> 3. Technical error: Eliminating possibilities 1 and 2 could point towards clustering issues the authors previously eluded

#Authors:
As we outlined above, (a) eCLIP data is human-only and much different than the Rfam's diverse set; (b) GraphClust2 doesn't have any explicit identity threshold and mainly relies on secondary structure level comparisons; (c) As shown in updated Fig.5-A, B, GraphClust2 has the same performance as the reference Rfam's CM. Rfam's CM is the ideal model since it has been built using the highly diverse set and covarying structures of Rfam's seed alignment.

> Would the authors be able to reconstruct the same secondary structure as in Rfam by using a simulated dataset composed of RF00032 sequences and noise?

#Authors:
We explicitly thank the reviewer for this suggestion. It helped to extend the evaluation and clarify a potential misinterpretation of the performance. Using RF00032 - 46 seed sequences combined with 2954 shuffled sequences of the same length and GC-content distribution. GraphClust2 was able to successfully predict the retrieve SLBP binding stem-loop with the same level of covariation as Rfam (new Fig. 5-B).

> Testing using real data:
> Another thing that I feel that needs to be answered is how well the tool is able to process a huge volume of real data. In a real case scenario, GraphClust2 would have to cluster millions of ncRNA sequences rather than just a few thousands mentioned in the paper. A possible dataset to benchmark the capabilities of the tool could be RNAcentral - the database of non-coding RNAs - currently containing almost 12 million sequences. This would raise the following questions:

#Authors:
We agree that demonstrating a very large scale dataset is a suitable add-on to the work. To answer this, we ran GraphClust2 on metatranscriptome dataset of ~3.6 million sequences and showed it's scalability and runtime linearity over the number of entries.
We would also like to highlight that structure-based clustering is a computationally-intense and cumbersome technique. We are not aware of any comparable tool (especially a publicly available & accessible one) that can de novo identification of even a thousand sequences. It should be also noted that identifying structural elements for the CLIP and lncRNAs are very demanding realistic scenarios and are quite large (e.g. XIST clustering was on 20,000 sequence fragments).

> 1. Would GraphClust2 be able to correctly classify the ncRNA sequences in their corresponding types?

#Authors:

While we agree that clustering and analyzing the entire RNAcental is an exciting study, we think that it is beyond the scope of this manuscript and deserves its own project. We believe that biologically novel and motivating questions should be first defined and prerequisites must be met (e.g. RNAcentral-Galaxy data interface), before investing such a large amount of effort on a project of that type. However, we hope we could convince the reviewer that GraphClust2 is scalable by analyzing a metatranscriptomics dataset with 3.6 Million of sequences.

> 2. Would the infrastructure be able to cope with such a dataset?

#Authors:
Yes. Galaxy and it's infrastructure is scalable to the degree that the computational resources behind Galaxy are scalable. The Europan Galaxy server has access to multiple clouds and HPC infrastructures, providing more than 5000 cores and 25TB of memory. We have performed the additional experiment on a very large dataset of transcriptomic sequences of marine community HTS data without facing any infrastructure challenge. Please refer to the new result section" Clustering runtime evaluation", where we have discussed the clustering of 3.6 million sequences from a metatranscriptomic sample, plus the new supplementary Fig. S1.

> 3. Graphclust2 runs on Galaxy platform via a GUI. What would the response time be in such cases?

#Authors:
We did not experience any lag or increase of the GUI response time while performing the million sequences metatranscriptome clustering. The interface response time is not expected to be affected by the computation load in a scalable Galaxy server. E.g. usegalaxy.eu is processing more than 150.000 jobs per month without any noticeable lag in response time.

> 4. Would the software be able to deal with noise that comes with real data?

#Authors:
The SLBP eCLIP and Roquin-1 PAR-CLIP data are both real data with an inherently large amount of noise. We have also shown that GraphClus2 can perform well with 98.5% noise of Rfam SLBP data. Again referring to the previous points, of course, biological interesting and narrowed-down questions and goals should be defined before answering this broad question.

> Technical issues with the dockerized version of GraphClust2:
> I was unable to pull the docker image and the command crashed with the error message "docker: unauthorized: authentication required." I tried a couple of probable solutions, but without any success. For this reason, I could not test the dockerized version of the tools and further testing would be required when the issue is resolved. It is important that the users do not experience this, especially when the software is targeting users with less technical expertise.

#Authors:
We are sorry that the reviewer could not use the docker instance. We have tested pulling the docker over several computers and different networks but have not faced the aforementioned problem. Searching the web hints for a generic issue about the mentioned error. It is likely related to the clock misconfiguration of the client login session or due to pulling a firewall/VPN. We would suggest trying an alternative (direct) internet connection and maybe also trying to logout and in the docker client again (using docker logout and login commands). Discussions and potential solutions are provided here: https://github.com/jupyter/docker-stacks/issues/364 and https://github.com/jupyter/docker-stacks/issues/484
A straightforward alternative option would be to run GraphClust2 on European Galaxy server under https://graphclust.usegalaxy.eu. We highlight that this server is running with strict data privacy policies such that the user data and activities are protected and not discernible. The usegalaxy.eu server is GDPR compliant and you can find the terms of use at https://usegalaxy.eu/terms/.

> Manuscript text:

> From the first read-through it becomes apparent that a good amount of effort went into the writing of the manuscript. However, although the manuscript is well structured, there were sections where rephrasing is essential for the text to be more readable. I also identified various linguistic errors as well as typos, so I would suggest another read-through to correct those. For example, there are a couple of typos on Figures 1 and 2. Figure 1 - I think the authors meant clustering instead of "clusteting" and on

Figure 2 - The title of the Y axis of both charts reads to "Adjusted Rand Inex" instead of Adjusted Rand Index, which is the term mentioned in the manuscript. I would also include the abbreviation ARI in parenthesis. I would also suggest avoiding strong words like "ultimate" and "superior" results (page 5).

#Authors:
We have read through the paper and revised the figures and resolved typos.

> Additionally, the background results should be analysed more thoroughly as these will give the users a better indication of how well GraphClust2 works and perhaps also provide answers to my previous questions.

#Authors:
We have also extended the discussions about the MALAT1 background data with discovery details. (Results, second paragraph of MALAT1)

> In addition to all the above, I also have the following suggestions and comments with respect to the tool usage and graphical user interface (GUI):

> 1. All the tools in GraphClust2 are accessible through a graphical user interface (GUI), which is dependent on the Galaxy Framework. On one hand this is nice, because it gives the users access to many other tools available through the Galaxy project, but limits the GraphClust usage to that. I believe that the provision of a command line version of the GraphClust2 toolset to enable batch processing of data would be very useful. This way the authors could also target more experienced users who prefer to use the CLIs of Linux based operating systems. Another benefit of a CLI compared to the current version of the GraphClust2 tools, is the parallelization of the data processing via utilization of HPC systems the users have access to. A command line version of the GraphClust2 toolset would also allow its integration with other systems as well

#Authors:
We agree that some users might feel more comfortable using a CLI and that is possible with Galaxy as well. Galaxy offers a RESTful API as well that can be targeted in various programming languages and that makes it possible to submit tools and workflows. However, we believe this is not the focus of the paper and is a more general Galaxy feature so we added a description of how GraphClust2 can be executed via a CLI in a new section of the readme on GitHub.
Parallelization is supported by Galaxy and the Docker Galaxy flavor which has GraphClust2 installed. In this sense, Galaxy is more or less just an abstraction layer to various HPC-schedulers and can be used to schedule jobs to your local HPC environment or Clouds.

> 2. GraphClust Galaxy tools: The names aren't very explanatory, and it was slightly hard to navigate the first time. The names of the shared workflows aren't descriptive either and it isn't very clear what each workflow corresponds to. The users can find useful documentation by visiting the GitHub repository, but in my opinion the two shouldn't be dependent on one another.

#Authors:
We have extended the content of the front page.

> The names of the individual tools are a bit confusing as well. A flowchart is provided in Figure 1, but I found it challenging trying to build a Galaxy workflow directly from that. One solution to this could be for the authors to include the names of the corresponding Galaxy tools in parenthesis, right within their matching component on the flowchart

#Authors:
The mapping between Figure 1 flowchart and matching Galaxy tools can be found within the GraphClust2 documentation and the front page of https://graphclust.usegalaxy.eu. Under the "GraphClust pipeline overview" section of the homepage, a number-annotated copy of Figure 1 is provided. The number-annotations are matched to the table at the bottom, where the components are described and linked.
Furthermore, we provide implemented galaxy workflow instances (based on the flowchart), such that a user can reuse our pre-build workflow and build-upon on that. The workflows and all results of this paper are linked from the front page.

> 3. GraphClust2 galaxy tour: I found the demo screen within the actual galaxy GraphClust screen confusing. When running in demo mode, from my experience with GraphClust2, Galaxy is basically mirroring the main webpage within the work panel of a tool with things overlapping. This makes the various demo steps hard to follow. I understand that sometimes issues like this one are due to framework limitations, but it would be nice if the authors could find an alternative solution to improve this

#Authors:
The html mirroring issue has been fixed, thanks for noticing.

Close
