# Supplementary material for: GraphClust2: Annotation and discovery of structured RNAs with scalable and accessible integrative clustering
Source: Gigascience. 2019 Dec 6;8(12):giz150. doi: 10.1093/gigascience/giz150 (PMC6897289; doi:10.1093/gigascience/giz150)
Supplement: giz150_Response_to_Reviewer_Comments_Revision_1 [file giz150_response_to_reviewer_comments_revision_1.pdf]

Author's Response To Reviewer Comments

Close

We would like to thank the reviewers for their feedback and the constructive reviewing process.

Close
