# Supplementary material for: GraphClust2: Annotation and discovery of structured RNAs with scalable and accessible integrative clustering
Source: Gigascience. 2019 Dec 6;8(12):giz150. doi: 10.1093/gigascience/giz150 (PMC6897289; doi:10.1093/gigascience/giz150)
Supplement: giz150_Response_to_Reviewer_Comments_Revision_2 [file giz150_response_to_reviewer_comments_revision_2.pdf]

Author's Response To Reviewer Comments

Close

Dear Scott,

We have fulfilled the requested revisions to the manuscript. We would like to thank the GigaScience editorial team and the reviewers for their constructive feedback and the opportunity for publishing our work in GigaScience.

On behalf of all authors,
Björn

Close
