# Supplementary material for: GraphClust2: Annotation and discovery of structured RNAs with scalable and accessible integrative clustering
Source: Gigascience. 2019 Dec 6;8(12):giz150. doi: 10.1093/gigascience/giz150 (PMC6897289; doi:10.1093/gigascience/giz150)
Supplement: giz150_Reviewer_1_Report_Original_Submission — Fabrizio Ferre -- 5/16/2019 Reviewed [file giz150_reviewer_1_report_original_submission.pdf]

Reviewer Report

Title: GraphClust2: annotation and discovery of structured RNAs with scalable and accessible integrative clustering

Version: Original Submission **Date:** 5/16/2019

Reviewer name: Fabrizio Ferre

Reviewer Comments to Author:

In the presented manuscript, the authors describe a tool for the clustering of RNAs based on secondary structure similarities. Their approach can find application in the classification of RNAs and in finding structural motifs. The method has stand-alone implementations as well as it is integrated within the Galaxy framework, with the aim of facilitating and standardizing its usage. While the manuscript is globally well written, some aspects of the method could be better clarified. The authors might want to consider the following points:

1. The clustering algorithm description is confusing. First, a graph kernel is used to identify RNAs forming initial clusters, which are then refined using UPGMA and CMfinder, and finally a covariance model is built for each cluster and used to scan the remaining RNAs. I don't understand how UPGMA and CMfinder are employed. Are they alternative to each other, or integrated in some undocumented way? Which information provided by UPGMA and/or CMfinder is used to compute the covariance model? Moreover, the iterative nature of the clustering algorithm is not evident from their description in the Materials and Methods section. I only realized that by looking at Fig. 2. I guess that the initial covariance models are progressively recomputed by adding new RNAs but, if it that's the case, a more detailed description of the procedure must be provided;
2. Is the LocARNA score an ultrametric? Can the graph kernel similarity scores be converted into a distance to feed UPGMA?
3. From the "Workflow output" section in M&M, it seems that fuzzy clusters (called soft clusters in the manuscript) can be obtained, but it is not explained how;
4. In the Results and Discussion, section "Locally conserved candidates…", manual checking and filtering is reported. I wonder which is the impact of these expert manual screens, and which results a non-expert could expect to obtain;
5. It is not clear how RNAz, Evofold and R-scape are used, whether they provide filtering criteria or are just used to annotate and describe the results;
6. Could running times for the described examples be provided?

Methods

Are the methods appropriate to the aims of the study, are they well described, and are necessary controls included? Choose an item.

Conclusions

Are the conclusions adequately supported by the data shown? Choose an item.

Reporting Standards

Does the manuscript adhere to the journal's guidelines on minimum standards of reporting? Choose an item.

Choose an item.

Statistics

Are you able to assess all statistics in the manuscript, including the appropriateness of statistical tests used? Choose an item.

Quality of Written English

Please indicate the quality of language in the manuscript: Choose an item.

Declaration of Competing Interests

Please complete a declaration of competing interests, considering the following questions:

- Have you in the past five years received reimbursements, fees, funding, or salary from an organisation that may in any way gain or lose financially from the publication of this manuscript, either now or in the future?
- Do you hold any stocks or shares in an organisation that may in any way gain or lose financially from the publication of this manuscript, either now or in the future?
- Do you hold or are you currently applying for any patents relating to the content of the manuscript?
- Have you received reimbursements, fees, funding, or salary from an organization that holds or has applied for patents relating to the content of the manuscript?
- Do you have any other financial competing interests?
- Do you have any non-financial competing interests in relation to this paper?

If you can answer no to all of the above, write 'I declare that I have no competing interests' below. If your reply is yes to any, please give details below.

I declare that I have no competing interests

I agree to the open peer review policy of the journal. I understand that my name will be included on my report to the authors and, if the manuscript is accepted for publication, my named report including any attachments I upload will be posted on the website along with the authors' responses. I agree for my report to be made available under an Open Access Creative Commons CC-BY license (http://creativecommons.org/licenses/by/4.0/). I understand that any comments which I do not wish to

be included in my named report can be included as confidential comments to the editors, which will not be published.

Choose an item.

To further support our reviewers, we have joined with Publons, where you can gain additional credit to further highlight your hard work (see: https://publons.com/journal/530/gigascience). On publication of this paper, your review will be automatically added to Publons, you can then choose whether or not to claim your Publons credit. I understand this statement.

Yes Choose an item.
