# Supplementary material for: GraphClust2: Annotation and discovery of structured RNAs with scalable and accessible integrative clustering
Source: Gigascience. 2019 Dec 6;8(12):giz150. doi: 10.1093/gigascience/giz150 (PMC6897289; doi:10.1093/gigascience/giz150)
Supplement: giz150_Reviewer_2_Report_Original_Submission — Ioanna Kalvari -- 5/27/2019 Reviewed [file giz150_reviewer_2_report_original_submission.pdf]

Reviewer Report

Title: GraphClust2: annotation and discovery of structured RNAs with scalable and accessible integrative clustering

Version: Original Submission **Date:** 5/27/2019

Reviewer name: Ioanna Kalvari

Reviewer Comments to Author:

The clustering of ncRNAs is an add-on to existing technologies of ncRNA annotation. This is done by allowing de-novo identification of ncRNA families and motifs, compared with the literature based family building process, starting from known ncRNA sequences which work as SEEDs. Also, tools that cluster ncRNA sequences are scarce and, in most cases, publicly unavailable, therefore projects such as this are essential. Building a ncRNA family requires a number of repetitive curation steps aiming to improve the initial multiple sequence alignment and consensus secondary structure. For that reason, tools that omit the expert contribution require thorough assessment.

The authors nicely demonstrated that the inclusion of structure probing data such as SHAPE, can improve the clustering performance of GraphClust2 when compared with its preceding version, namely GraphClust. However, the results of the experiment based on eCLIP data left me questioning the quality of the clustering methodology and the test dataset.

The secondary structure generated from the largest cluster from the eCLIP data experiment, shows loss in base-pair covariation compared with the consensus secondary structure obtained from Rfam. It is very important to ensure the clustering works efficiently enough, as base-pair covariation is evidence that the secondary structure of a family of ncRNAs is correct.

The following points could help investigate this further:

1. How taxonomically diverse is the dataset used? Although the dataset apart from human sequences also includes sequences from other species - which the authors do not mention in the manuscript - is likely not diverse enough. Histone3 family (RF00032) is built from 46 sequences coming from 28 distinct species
2. What is the sequence identity threshold and how was it decided for the best clustering results? This is something the authors did not mention in the manuscript and testing different thresholds could potentially result in gain of base-pair covariation support
3. Technical error: Eliminating possibilities 1 and 2 could point towards clustering issues the authors previously eluded

Would the authors be able to reconstruct the same secondary structure as in Rfam by using a simulated dataset composed of RF00032 sequences and noise?

Testing using real data:

Another thing that I feel that needs to be answered is how well the tool is able to process a huge volume of real data. In a real case scenario, GraphClust2 would have to cluster millions of ncRNA sequences rather than just a few thousands mentioned in the paper. A possible dataset to benchmark the capabilities of the tool could be RNAcentral - the database of non-coding RNAs - currently containing

almost 12 million sequences. This would raise the following questions:
1. Would GraphClust2 be able to correctly classify the ncRNA sequences in their corresponding types?
2. Would the infrastructure be able to cope with such a dataset?
3. Graphclust2 runs on Galaxy platform via a GUI. What would the response time be in such cases?
4. Would the software be able to deal with noise that comes with real data?

Technical issues with the dockerized version of GraphClust2:

I was unable to pull the docker image and the command crashed with the error message "docker: unauthorized: authentication required." I tried a couple of probable solutions, but without any success. For this reason, I could not test the dockerized version of the tools and further testing would be required when the issue is resolved. It is important that the users do not experience this, especially when the software is targeting users with less technical expertise.

Manuscript text:

From the first read-through it becomes apparent that a good amount of effort went into the writing of the manuscript. However, although the manuscript is well structured, there were sections where rephrasing is essential for the text to be more readable. I also identified various linguistic errors as well as typos, so I would suggest another read-through to correct those. For example, there are a couple of typos on Figures 1 and 2. Figure 1 - I think the authors meant clustering instead of "clusteting" and on Figure 2 - The title of the Y axis of both charts reads to "Adjusted Rand Inex" instead of Adjusted Rand Index, which is the term mentioned in the manuscript. I would also include the abbreviation ARI in parenthesis. I would also suggest avoiding strong words like "ultimate" and "superior" results (page 5). Additionally, the background results should be analysed more thoroughly as these will give the users a better indication of how well GraphClust2 works and perhaps also provide answers to my previous questions.

In addition to all the above, I also have the following suggestions and comments with respect to the tool usage and graphical user interface (GUI):

1. All the tools in GraphClust2 are accessible through a graphical user interface (GUI), which is dependent on the Galaxy Framework. On one hand this is nice, because it gives the users access to many other tools available through the Galaxy project, but limits the GraphClust usage to that. I believe that the provision of a command line version of the GraphClust2 toolset to enable batch processing of data would be very useful. This way the authors could also target more experienced users who prefer to use the CLIs of Linux based operating systems. Another benefit of a CLI compared to the current version of the GraphClust2 tools, is the parallelization of the data processing via utilization of HPC systems the users have access to. A command line version of the GraphClust2 toolset would also allow its integration with other systems as well
2. GraphClust Galaxy tools: The names aren't very explanatory, and it was slightly hard to navigate the first time. The names of the shared workflows aren't descriptive either and it isn't very clear what each workflow corresponds to. The users can find useful documentation by visiting the GitHub repository, but in my opinion the two shouldn't be dependent on one another. The names of the individual tools are a bit confusing as well. A flowchart is provided in Figure 1, but I found it challenging trying to build a Galaxy workflow directly from that. One solution to this could be for the authors to include the names of the corresponding Galaxy tools in parenthesis, right within their matching component on the flowchart
3. GraphClust2 galaxy tour: I found the demo screen within the actual galaxy GraphClust screen

confusing. When running in demo mode, from my experience with GraphClust2, Galaxy is basically mirroring the main webpage within the work panel of a tool with things overlapping. This makes the various demo steps hard to follow. I understand that sometimes issues like this one are due to framework limitations, but it would be nice if the authors could find an alternative solution to improve this

Methods

Are the methods appropriate to the aims of the study, are they well described, and are necessary controls included? Choose an item.

Conclusions

Are the conclusions adequately supported by the data shown? Choose an item.

Reporting Standards

Does the manuscript adhere to the journal's guidelines on minimum standards of reporting? Choose an item.

Choose an item.

Statistics

Are you able to assess all statistics in the manuscript, including the appropriateness of statistical tests used? Choose an item.

Quality of Written English

Please indicate the quality of language in the manuscript: Choose an item.

Declaration of Competing Interests

Please complete a declaration of competing interests, considering the following questions:

- Have you in the past five years received reimbursements, fees, funding, or salary from an organisation that may in any way gain or lose financially from the publication of this manuscript, either now or in the future?
- Do you hold any stocks or shares in an organisation that may in any way gain or lose financially from the publication of this manuscript, either now or in the future?
- Do you hold or are you currently applying for any patents relating to the content of the manuscript?
- Have you received reimbursements, fees, funding, or salary from an organization that holds or has applied for patents relating to the content of the manuscript?
- Do you have any other financial competing interests?

- Do you have any non-financial competing interests in relation to this paper?

If you can answer no to all of the above, write 'I declare that I have no competing interests' below. If your reply is yes to any, please give details below.

I declare that I have no competing interests

I agree to the open peer review policy of the journal. I understand that my name will be included on my report to the authors and, if the manuscript is accepted for publication, my named report including any attachments I upload will be posted on the website along with the authors' responses. I agree for my report to be made available under an Open Access Creative Commons CC-BY license (http://creativecommons.org/licenses/by/4.0/). I understand that any comments which I do not wish to be included in my named report can be included as confidential comments to the editors, which will not be published.

Choose an item.

To further support our reviewers, we have joined with Publons, where you can gain additional credit to further highlight your hard work (see: https://publons.com/journal/530/gigascience). On publication of this paper, your review will be automatically added to Publons, you can then choose whether or not to claim your Publons credit. I understand this statement.

Yes Choose an item.
