# Supplementary material for: GraphClust2: Annotation and discovery of structured RNAs with scalable and accessible integrative clustering
Source: Gigascience. 2019 Dec 6;8(12):giz150. doi: 10.1093/gigascience/giz150 (PMC6897289; doi:10.1093/gigascience/giz150)
Supplement: giz150_Reviewer_2_Report_Revision_1 — Ioanna Kalvari -- 10/14/2019 Reviewed [file giz150_reviewer_2_report_revision_1.pdf]

Reviewer Report

Title: GraphClust2: annotation and discovery of structured RNAs with scalable and accessible integrative clustering

Version: Revision 1 **Date:** 10/14/2019

Reviewer name: Ioanna Kalvari

Reviewer Comments to Author:

The authors have responded to all of the questions and provided additional information and clarifications where necessary. The authors also performed new experiments to expand and strengthen the indicated sections, resolved any technical issues and modified the text according to the suggestions. I do not have anything further to add apart from reporting some minor text errors I identified with a second read-through.

The following is a list of points I identified in the text that require editing:

1. Intro, page 2 at the end of the section where the authors list their goals "(v) allowing for efficient parallelizations over different platforms;" - I believe that sentence should followed by a '.'.
2. Similar to 1 in Materials and methods, first paragraph after "(v) cluster collection, visualization and annotation;" ; should be replaced with .
3. These repeats 4 times in the newly added text that follows

a. After "specific tools; Firstly, from..."

b. After "smaller than the entire input data; In the second step after..."

c. After "maximization algorithm [28]; Thirdly after..". Perhaps a comma is also needed after "Thirdly"?

d. After "missed in the initial clustering; Finally, ..."

4. In the workflow output paragraph: I believe ending the sentence after "soft partitions" and starting with a new sentence as "soft partitions. In the latter case, elements can belong..." makes the text easier to read.
5. A full stop is needed after subtitle " Orthology sequence extraction from long RNA locus"
6. Page 5, end of column 1 - "To draw a conclusion, however, an extensive SHAPE data would be needed...". Would "To draw a conclusion, however, an extensive SHAPE dataset would be needed.." or "To draw a conclusion, however, extensive SHAPE data would be needed..." be more appropriate here?
7. There is a full stop at the end of title "GraphClust is Scalable." Delete if not purposefully used there
8. Page 6, column 2, right before the FTL paragraph. "And compensatory mutated paired regions; Below we describe..." Replace ; with .
9. Page 7 - The first sentence of the newly added text requires a full stop.

I would encourage the authors to do another read-through of the manuscript in case I missed something.

I am also attaching a doc file, with my full responses (blue colour) to the author comments.

Methods

Are the methods appropriate to the aims of the study, are they well described, and are necessary controls included? Choose an item.

Conclusions

Are the conclusions adequately supported by the data shown? Choose an item.

Reporting Standards

Does the manuscript adhere to the journal's guidelines on minimum standards of reporting? Choose an item.

Choose an item.

Statistics

Are you able to assess all statistics in the manuscript, including the appropriateness of statistical tests used? Choose an item.

Quality of Written English

Please indicate the quality of language in the manuscript: Choose an item.

Declaration of Competing Interests

Please complete a declaration of competing interests, considering the following questions:

- Have you in the past five years received reimbursements, fees, funding, or salary from an organisation that may in any way gain or lose financially from the publication of this manuscript, either now or in the future?
- Do you hold any stocks or shares in an organisation that may in any way gain or lose financially from the publication of this manuscript, either now or in the future?
- Do you hold or are you currently applying for any patents relating to the content of the manuscript?
- Have you received reimbursements, fees, funding, or salary from an organization that holds or has applied for patents relating to the content of the manuscript?
- Do you have any other financial competing interests?
- Do you have any non-financial competing interests in relation to this paper?

If you can answer no to all of the above, write 'I declare that I have no competing interests' below. If your reply is yes to any, please give details below.

I declare that I have no completing interests

I agree to the open peer review policy of the journal. I understand that my name will be included on my report to the authors and, if the manuscript is accepted for publication, my named report including any attachments I upload will be posted on the website along with the authors' responses. I agree for my report to be made available under an Open Access Creative Commons CC-BY license (http://creativecommons.org/licenses/by/4.0/). I understand that any comments which I do not wish to be included in my named report can be included as confidential comments to the editors, which will not be published.

Choose an item.

To further support our reviewers, we have joined with Publons, where you can gain additional credit to further highlight your hard work (see: https://publons.com/journal/530/gigascience). On publication of this paper, your review will be automatically added to Publons, you can then choose whether or not to claim your Publons credit. I understand this statement.

Yes Choose an item.
